# Acculturation and Depressive Symptoms among Turkish Immigrants in Germany 

**DOI:** 10.3390/ijerph110909503

**Published:** 2014-09-12

**Authors:** Eva Morawa, Yesim Erim

**Affiliations:** Department of Psychosomatic and Psychotherapeutic Medicine, Friedrich-Alexander University Erlangen-Nürnberg (FAU), Schwabachanlage 6, 91054 Erlangen, Germany; E-Mail: yesim.erim@uk-erlangen.de

**Keywords:** immigrants, Turkish, acculturation, mental health, depressive symptoms

## Abstract

The present study explores the impact of acculturation on depressive symptoms among Turkish immigrants in Germany, taking into account different dimensions of cultural orientation. A total of 471 patients from two selected samples (254 primary care patients and 217 outpatients of a psychosomatic department) participated. Levels of acculturation were measured as orientation towards culture of origin (CO), and orientation towards the host culture (HC). Acculturation strategies (integration, assimilation, separation, and marginalization) were also assessed as well as their association with depressive symptoms (BDI). Furthermore, gender- and migration-related differences in terms of acculturation and levels of depressive symptomatology were analyzed. Integration was the acculturation strategy associated with the lowest level of depressive symptoms (M = 14.6, SD = 11.9), while marginalization was associated with the highest (M = 23.5, SD = 14.7). Gender was not found to have a significant impact on acculturation but influenced depressive symptoms, with women (M = 21.8, SD = 13.3) reporting higher levels of depressive symptomatology than men (M = 15.1, SD = 14.0; *p* < 0.001). In first generation immigrants, significantly higher CO (M = 46.6, SD = 8.3; *p* < 0.001), lower HC (M = 31.0, SD = 9.6; *p* < 0.001), and higher levels of depressive symptoms (M = 20.2, SD = 14.1; *p* < 0.001) were found in comparison to second generation immigrants (CO: M = 41.3, SD = 7.4; HC: M = 36.2, SD = 8.8; depressive symptoms: M = 14.0, SD = 12.9). Our results suggest that orientation towards both the heritage and the host culture has a positive effect on the mental health status of immigrants. Future research needs to include representative samples of migrants from different cultures to further explore the association between acculturation and mental health.

## 1. Introduction

Over the last decades, a worldwide increase in international migration has been observed. Europe is the region with the highest rate of immigrants, with 8.7% of the total migration population and every third migrant worldwide living in this continent [1]. In Germany, every fifth person has a migration background, either having personally moved there or having at least one parent who moved [2].

In the face of expanding numbers of immigrants, scientific interest on migration and its impact on mental health has increased in recent years. Empirical findings are controversial [3,4], however, most studies have reported higher mental distress among immigrants compared to the indigenous population [5,6].

Not only is the immigration process itself associated with challenging demands, but the post-migration acculturation process can also have an influence on health status. Acculturation is a complex, multidimensional, and long-term process of psychological and social changes resulting from continuous interaction between individuals from different cultures [7]. These changes include learning a new language, creating a new social network, integrating new values, beliefs, attitudes, lifestyle patterns and so on.

With respect to acculturation, the most renowned theory is the one by Berry [8], which proposes four acculturation strategies: integration, assimilation, separation, and marginalization. The majority of studies on acculturation styles and mental health have so far been conducted in the United States [9]. To the best of our knowledge, Berry’s theory has not yet been investigated among Turkish migrants living in Germany. Our study is therefore the first to explore the relationship between the four acculturation types and depressive symptoms, among Germany’s largest immigration group.

Most empirical evidence demonstrates integration to be the most successful strategy, as it is associated with the lowest acculturation stress levels and demonstrates good health outcomes, whereas marginalization seems to be the most stress-related strategy, with assimilation and separation having intermediate positions [8,9].

However, there have also been some contradictory findings reported. For example, some higher acculturated immigrants have described more symptoms of depression than less acculturated individuals [10]. One explanation for the inconsistent results could be that acculturation consists of various aspects (e.g., language being the most important), and there is no mutual agreement on conceptualization and measurement of the phenomenon of acculturation. In addition, confounding variables such as socio-economic status and discrimination for example, do not allow for a clear relationship between acculturation and mental health to be established [11].

### 1.1. Immigrants of Turkish Origin in Germany

In West Germany, World War II and the economic boom of the 1950s and 1960s led to a shortage of laborers and as a result the government signed recruitment agreements with different countries such as Italy (1955), Greece (1960), Spain (1960), Turkey (1961), Portugal (1964) and Yugoslavia (1968). The so called “guest workers” (“Gastarbeiter”) were mainly less educated, unskilled laborers from rural areas who were recruited for elementary jobs in factories and were expected to return to their home country after a few years. Between 1968 and 1973, the percentage of Turkish people in relation to all guest workers coming to Germany had risen from 10.7% to 23.0% and after 1971, persons of Turkish origin were the largest group of guest workers [12]. Due to the petroleum crisis in 1973, the recruitment of guest workers was discontinued and following this the only way to enter Germany was on the basis of family reunification. As a result, Turkish immigration into Germany changed from temporary, work-related, and male-dominated, to long-term and family-oriented [13]. Political instability in Turkey at the end of the 1970s and beginning of the 1980s caused a second flow of immigration in terms of asylum seekers (as well as intellectuals). Currently, individuals of Turkish origin (2,998,000 persons) represent the largest immigration group and constitute almost one-fifth (18.3%) of all persons living in Germany with a migration background [2]. Contrastingly, Turkish migration to North America has been characterized by smaller numbers [14,15] and often with a higher socio-economic status, such as professionals and students [14].

### 1.2. Acculturation and Mental Health in Turkish Immigrants

Studies on acculturation and depression or other indicators of mental health in Turkish immigrants living in Germany or other European countries, have shown inconsistent results. Gunay and Haag [16] reported higher levels of psychiatric symptoms indicating depressive mood states (e.g., loss of interest) in lesser acculturated compared to higher acculturated Turkish women of a primary care unit, but similar values with regard to somatic complaints. No significant differences were found between higher and less acculturated Turkish women at a gynaecological unit in Berlin for eight of nine subscales of the Symptom Checklist Questionnaire (SCL-90-R) [17]. Tydecks *et al.* [18] examined 100 Turkish patients of two general physicians’ practices in Berlin with the SCL-14 (Center for Epidemiologic Studies Depression Scale, CES-D); and a questionnaire assessing the acculturation level (FRACC), which was also applied in the present study. Probands with higher acculturation values presented significantly lower depression levels when controlled for gender, but this association disappeared for those men demonstrating even higher acculturation levels than the women.

Fassaert *et al.* [19] assessed acculturation and psychological distress among 321 first generation Muslim immigrants from Turkey and Morocco, living in The Netherlands. In the Turkish sample, a reduced ability to adapt to Dutch society, largely relating to poor language proficiency, was found to be associated with higher levels of psychological distress. In addition, it could be observed that men were subjected to less distress when conservative values and norms were applied. Baltas and Steptoe [20] found no significant association between acculturation (assessed as maintenance of Turkish traditions) and psychological well-being (measured as BDI “depression” and STAI “anxiety”) among a sample of Turkish immigrants living in the UK. In a study of 407 adolescents with a Turkish migration background who were living in Norway and Sweden [21], Turkish identity and the acculturation strategy of integration were predictors of good psychological adaptation (mental health problems, self-esteem and life satisfaction), whereas poor adaptation was predicted by marginalization and perceived discrimination.

### 1.3. Acculturation and Mental Health in Other Immigrant Populations

Investigations on acculturation and depression in other migrant populations have provided empirical support for the protective function of adaptation to a new culture on the mental health of immigrants. A meta-analysis on the relationship between acculturation and depression among Asian Americans [22], based on 38 studies, found acculturation (assessed as “assimilation to the American culture”) to be significantly negatively associated with depression; while acculturation (assessed as “orientation to the Asian culture”) was also negatively associated, but not in a statistically significant way. In a study conducted in the USA consisting of 172 Korean immigrants [23], no significant differences with regard to depressive symptoms (CES-D) between high and low orientation to the host culture or high and low orientation to the heritage culture were observed, however, significant higher scores on positive affect items were found in the group of subjects who were higher acculturated to the American culture. In a study of Polish and Vietnamese immigrants in Germany [24], a high identification with Germany was a significant predictor of low levels of depression and for the Polish sample, so were a high social assimilation, and good language skills.

### 1.4. Measures of Acculturation

Two main methods are applied in investigations to measure acculturation: proxy and scaled measures [22]. Proxy measures performed as either a single item (e.g., language proficiency), or a few items, is an indirect and often preferred method because of its efficiency. In contrast, scaled measures are often based on a theory and consist of a greater range of features. They can be categorized into three types: unidimensional, bidimensional and multidimensional [25]. Unidimensional instruments describe acculturation as a continuum ranging from unacculturated to acculturated, postulating assimilation to the majority culture as the index of acculturation. Bidimensional instruments use two separate scales to examine the level of maintenance to the culture of origin and the level of identification with the new culture. Multidimensional instruments explore multiple elements of acculturation such as values, attitudes and so on, with separate scales.

### 1.5. Depression

Depression is a mood disorder characterized by low mood and loss of interest which are accompanied by other cognitive, somatic and behavioral symptoms [26]. Depression occurs worldwide and is one of the most frequent and cost-intensive mental disorders. It is highly prevalent among the general population, for example in Germany the lifetime prevalence rate for any mood disorder is 18.6% [27]. Among immigrants, the prevalence rates for depression lie between 3% and 47% for labor migrants, and between 3% and 81% for refugees [28]. The occurrence of depressive disorders among women and female migrants is twice as high as among men [27] and male migrants [3]. Depression seems to be a universal phenomenon, however significant differences in terms of its clinical presentation can be observed across different cultures [26]. Variations in symptom severity have also been reported. Patients of Turkish origin show higher levels of depressive symptoms in comparison with indigenous patients [29].

### 1.6. Study Aim

The main aim of the present study was to investigate the association between acculturation and depressive symptoms among Turkish immigrants in Germany. Acculturation was analyzed using a bi-dimensional approach (identification with the culture of origin “CO”, and identification with the host culture “HC”) as well as by applying a categorization of four acculturation strategies (integration, assimilation, separation, and marginalization).

The following questions were examined:

(1) Is there an association between the acculturation strategy (integration, assimilation, separation, and marginalization) and level of depressive symptoms?
(2) Are variations to be found in the degree of acculturation and depressive symptoms according to gender and migrant generation level (first generation immigrants = born in Turkey *vs.* second generation immigrants = born in Germany)?
(3) What are socio-demographic, migration and acculturation-related predictors in determining the level of depressive symptoms among Turkish immigrants?

Based on previous studies, we hypothesized integration to be the acculturation strategy showing the lowest levels of depressive symptoms and marginalization to be the strategy showing the highest. We expected women and first generation immigrants to demonstrate higher scores on depressive symptoms, higher CO and lower HC than men and second generation immigrants, respectively. Lastly, we postulated that both CO and HC would be significant negative predictors of the level of depressive symptoms.

## 2. Methods

### 2.1. Participants

Participants were recruited consecutively from the Turkish-language Outpatient Unit, established in 1995, at the Department of Psychosomatic Medicine and Psychotherapy in Essen (North Rhine-Westphalia). This service is available to patients who either do not have sufficient knowledge of the German language to be able to convey their complaints, or who wish to speak to someone acquainted with their cultural background.

Further recruitment took place at the practice of a general practitioner, whose mother tongue is Turkish. This particular practice has existed for 25 years and is located in an area that has one of the highest rates of persons of Turkish background compared to other migrant populations in Essen. Every third patient of Turkish origin who came into the practice was recruited.

Inclusion criteria for the study were: age of consent (minimum of 18 years), agreement to participate in the study, and the status of a person of Turkish descent according to the definition as applied in epidemiological research in Germany [30], that is, having either immigrated themselves or having at least one parent who immigrated. Exclusion criteria were diagnoses of either psychosis, organic brain disorders, or poor aptitude.

In the general practitioner practice the response rate was 93.2%, and in the Turkish-language Outpatient Unit it was 85.3%. After excluding two psychosomatic outpatients on the basis of exclusion criteria, and 13 psychosomatic outpatients on the basis of too many missing values in the questionnaires (no primary care patients had to be excluded), a total sample of 254 primary care patients and 217 psychosomatic outpatients of Turkish origin, were included in the study.

### 2.2. Procedure and Setting

Data were collected consecutively between August 2011 and January 2013 in the above mentioned recruitment centers. Patients of the Turkish-language Outpatient Unit at the Department of Psychosomatic Medicine and Psychotherapy in Essen were given appointments for an initial interview and a written explanation of the study in which they were also asked to participate. Primary care patients were asked to participate in the study when they registered by a Turkish-speaking doctoral candidate, who then provided them with a written explanation of the study. Participants either completed the self-report questionnaire at home and brought it with them to the initial interview (patients of the Turkish-language outpatient clinic), or completed it in a separate room at the general physician’s practice (primary care patients) that had been especially prepared for the study. Questions concerning individual items were answered either by Turkish-speaking psychologists at the initial interview (outpatient clinic) or by the doctoral candidate (general physician’s practice).

### 2.3. Ethics Statement

The present cross-sectional study was conducted in accordance with the Declaration of Helsinki, and the protocol was approved by the Ethics Committee of the Medical Faculty of the University of Duisburg-Essen (Project identification code: 11-4883-BO). All subjects gave their informed consent for inclusion before they participated in the study.

### 2.4. Measures

#### 2.4.1. Socio-Demographic and Migration-Specific Variables

The following socio-demographic and migration-specific items were assessed: gender, age, marital status, education level, employment status, income, length of residence in Germany, age at time of immigration into Germany, reason for immigration, and language proficiency.

#### 2.4.2. Depressive Symptoms

Depressive symptoms were measured by the Beck Depression Inventory [31], which consists of 21 items representing the most important symptoms of depression. Scores range between 0 and 63. A total score of 18 points and above indicates a clinically relevant depression. In this study a validated Turkish version of the BDI [32] was employed, which obtained a Cronbach’s Alpha of 0.94 in the present sample.

#### 2.4.3. Acculturation

The acculturation level was assessed with the Frankfurt Acculturation Scale (FRACC) [33], a self-report questionnaire with 20 items rated on a seven-point Likert scale (0 = absolutely not to 6 = absolutely). The inventory consists of two indices measuring the degree of orientation towards culture of origin (CO), and the level of orientation towards the host culture (HC), using 10 items and with values between 0 and 60 for each index. Higher scores indicate a higher orientation towards the CO or HC, respectively. A Turkish version of the FRACC was used in the current study, with a Cronbach’s Alpha of 0.72 for CO and 0.71 for HC.

**Figure 1:**
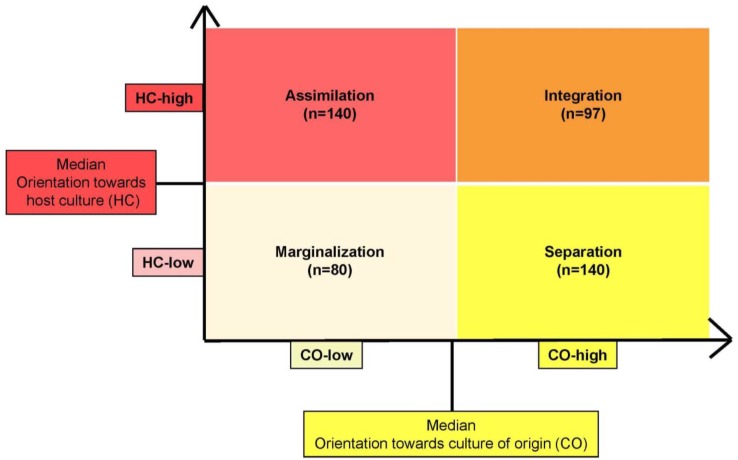
Distribution of the study population in the acculturation strategies based on the model of Berry [7].

The medians of both indices of the acculturation scale were used as cutoff points to divide the sample into a group of subjects with a low CO *vs.* a group with a high CO, as well as a group of subjects with a low HC *vs.* a group with a high HC. We categorized the probands into four groups (= acculturation strategies) based on the schema of Berry [7]: integration (CO-high and HC-high), assimilation (HC-high and CO-low), separation (CO-high and HC-low), and marginalization (CO-low and HC-low); see Figure 1.

### 2.5. Statistical Analysis

Data analyses were carried out by means of SPSS V. 21 (IBM Deutschland GmbH, Ehningen, Germany). The following descriptive statistics of the socio-demographic and migration-specific variables were computed: means, standard deviations, ranges and frequencies. Missing values in the questionnaires were replaced by the rounded mean of the fulfilled items of the sub-scales (max. 20% missing data accepted). For categorical data, parametric tests (χ^2^-test or Fisher’s exact test) were performed; for continuous data, t-tests for one sample and independent samples were computed. In addition, we carried out ANOVAS with the Bonferroni test as a *post-hoc*-test, correlation analyses according to Pearson and Spearman, as well as linear step-wise regression analyses. A level of significance of *p* < 0.05 was predetermined.

## 3. Results

### 3.1. Socio-Demographic Data

The socio-demographic characteristics of participants are presented in Table 1. The total sample (N = 471; 53.7% women) consisted of 254 primary care patients and 217 outpatients of a psychosomatic unit, with both groups being of Turkish descent (Table 1). The average age at the time of the study was 39.7 years (SD = 11.5). Overall, 70.7% of the participants were married, 43.5% were employed, 39.1% had a secondary/vocational education and 29.5% had a monthly household income of between €1000–2000. For all the socio-demographic data examined, significant differences were found between the two sub-groups.

**Table 1:** Demographic characteristics of the study sample.

Socio-Demographic Variables		Total (*N* = 471)	Primary Care Patients of Turkish Origin (*n* = 254)	Psychosomatic Outpatients of Turkish Origin (*n* = 217)	*p*-values
Gender	women	253 (53.7%)	109 (42.9%)	144 (66.4%)	*p* < 0.001
	men	218 (46.3%)	145 (57.1%)	73 (33.6%)	
Age	mean (SD)	39.7 (11.5)	38.4 (12.3)	41.2 (10.4)	*p* = 0.006
	range	18–78	18–78	18–73	
Marital status	single	67 (14.2%)	46 (18.1%)	21 (9.7%)	*p* < 0.001
	married	333 (70.7%)	190 (74.8%)	143 (65.9%)	
	widowed	20 (4.2%)	8 (3.1%)	12 (5.5%)	
	separated/divorced	51 (10.8%)	10 (3.9%)	41 (18.9%)	
Education	no education	15 (3.2%)	6 (2.4%)	9 (4.1%)	*p* < 0.001
	primary school	125 (26.5%)	45 (17.7%)	80 (36.9%)	
	middle school	104 (22.1%)	66 (26.0%)	38 (17.5%)	
	secondary/vocational school	184 (39.1%)	106 (41.7%)	78 (35.9%)	
	university/university of applied technology	43 (9.1%)	31 (12.2%)	12 (5.5%)	
Employment status	employed	205 (43.5%)	140 (55.1%)	65 (30.0%)	*p* < 0.001
	unemployed (household)	112 (23.8%)	40 (15.7%)	72 (33.2%)	
	jobless	52 (11.0%)	19 (7.5%)	33 (15.2%)	
	pensioner	41 (8.7%)	25 (9.8%)	16 (7.4%)	
	pupil/student	35 (7.4%)	26 (10.2%)	9 (4.1%)	
	on sick leave	26 (5.5%)	4 (1.6%)	22 (10.1%)	
Monthly household income	<€500	70 (14.9%)	28 (11.0%)	42 (19.4%)	*p* = 0.002
	€500–1000	116 (24.6%)	61 (24.0%)	55 (25.3%)	
	€1000–2000	139 (29.5%)	66 (26.0%)	73 (33.6%)	
	€2000–3000	50 (10.6%)	38 (15.0%)	12 (5.5%)	
	>€3000	21 (4.5%)	13 (5.1%)	8 (3.7%)	
	no data	75 (15.9%)	48 (18.9%)	27 (12.4%)	

### 3.2. Migration-Specific Data

Table 2 reports the migration-related data of the study sample. The average duration of residence of Turkish patients in Germany was 24.3 years (SD = 11.1) at the time of data collection (Table 2).

**Table 2:** Migration-specific characteristics of the study sample.

Migration-Specific Variables		Total (*N* = 471)	Primary Care Patients of Turkish Origin (*n* = 254)	Psychosomatic Outpatients of Turkish Origin (*n* = 217)	*p*-values
Length of residence in Germany	mean (SD)	24.3 (11.1)	25.8 (10.9)	23.0 (11.0)	*p* = 0.014
	range	<1–48	<1–46	1–48	
Age at immigration	mean (SD)	18.9 (8.0)	17.8 (7.8)	19.9 (8.1)	*p* = 0.012
	range	<1–46	1–40	<1–46	
Reason for immigration	born in Germany	108 (22.9%)	82 (32.3%)	26 (12.0%)	*p* < 0.001
	family reunion	106 (22.5%)	68 (26.8%)	38 (17.5%)	
	marriage	184 (39.1%)	67 (26.4%)	117 (53.9%)	
	work	35 (7.4%)	19 (7.5%)	16 (7.4%)	
	study	7 (1.5%)	7 (2.8%)	0	
	political reasons	16 (3.4%)	6 (2.4%)	10 (4.6%)	
	other	6 (1.3%)	2 (0.8%)	4 (1.8%)	
	no data	9 (1.9%)	3 (1.2%)	6 (2.8%)	
Language proficiency	German as mother tongue	26 (5.5%)	20 (7.9%)	6 (2.8%)	*p* < 0.001
	very good	53 (11.3%)	41 (16.1%)	12 (5.5%)	
	good	121 (25.7%)	83 (32.7%)	38 (17.5%)	
	moderate	179 (38.0%)	86 (33.9%)	93 (42.9%)	
	little	90 (19.1%)	24 (9.4%)	66 (30.4%)	
	none	2 (0.4%)	0	2 (0.9%)	

The immigrants were, on average, 18.9 (SD = 8.0) years old at the time of their arrival in Germany, and 22.9% were born in Germany. For the largest part of the sample (39.1%), marriage was the reason for immigration (for persons from non-EU countries marriage to a German citizen is one of the few legal reasons for being admitted to Germany). The language proficiency was rated as moderate by most (38.0%) of the investigated subjects.

### 3.3. Acculturation Strategies (Integration, Assimilation, Separation, and Marginalization) and Depression Levels

An ANOVA comparing levels of depressive symptoms for each acculturation strategy in the total sample demonstrated significant differences between the strategies (F = 8.56, *p* < 0.001). Integration (M = 14.6, SD = 11.9) was indicated to be the strategy with the lowest level of depressive symptoms and marginalization (M = 23.5, SD = 14.7) was the strategy with the highest level.

**Figure 2:**
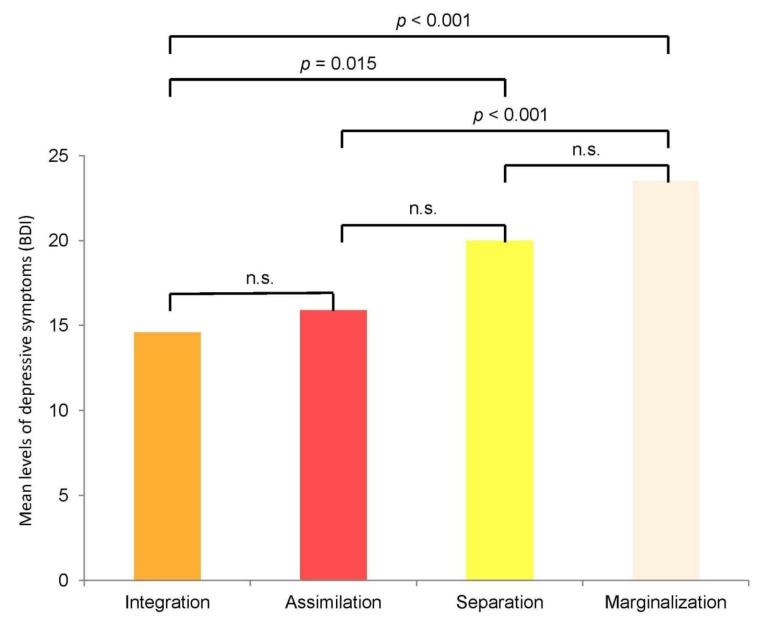
Mean levels of depressive symptoms (BDI) according to acculturation strategies.

Note: n.s. = not significant.

Integration was associated with significantly lower degrees of depressive symptomatology than marginalization (*p* < 0.001) and separation (M = 20.0, SD = 13.6; *p* = 0.015), and also had lower values than assimilation but not to a significant level (M = 15.9, SD = 13.3; *p* = 1.0). Assimilation demonstrated significantly lower scores for depressive symptomatology in comparison to marginalization (*p* < 0.001) and a trend for lower values compared to separation (*p* = 0.073). Separation and marginalization did not differ significantly (*p* = 0.414; see Figure 2).

### 3.4. Acculturation (CO and HC) and Depression Levels according to Gender, Migrant Generation, and Setting

For the total sample, as well as the sub-samples of primary care patients and psychosomatic patients, no significant gender-specific differences with regard to the levels of CO and HC were found. However, the generational status had an impact on both acculturation indices in the total sample and in both subgroups. The first migration generation (*i.e.*, persons born in Turkey) demonstrated significantly (*p* < 0.001) higher levels of CO than the second generation (*i.e.*, individuals born in Germany) in the total sample as well as both subgroups, whereas the second generation was significantly more acculturated to the German culture (total sample: *p* < 0.001; primary care patients: *p* = 0.001; psychosomatic patients: *p* = 0.004) than was the case for the first generation immigrants.

In terms of severity of depressive symptoms, significant differences were found in the total sample between both migration generations and also between the genders. Participants who were born in Turkey (M = 20.2, SD = 14.1) reported significantly higher levels of depressive symptomatology than persons with a migration background but born in Germany (M = 14.0, SD = 12.9; *p* < 0.001); women (M = 21.8, SD = 13.3) had a significantly higher mean score than men (M = 15.1, SD = 14.0; *p* < 0.001, see Table 3). This pattern was also observed for primary care patients, while psychosomatic patients did not differ significantly.

When analyzing items of the Beck Depression Scale separately, the items “irritability”, “fatigability” and “work difficulty” showed the highest mean value for the total sample (M = 1.3, SD = 1.1; M = 1.3, SD = 0.9; M = 1.2, SD = 1.0), as well as for women (M = 1.5, SD = 1.1; M = 1.5, SD = 0.9; M = 1.4, SD = 1.0) *vs.* men (M = 1.1, SD = 1.1, *p* < 0.001; M = 1.0, SD = 0.9, *p* < 0.001; M = 1.0, SD = 1.0, *p* < 0.001), and for individuals of the first migration generation (M = 1.4, SD = 1.1; M = 1.4, SD = 0.9; M = 1.3, SD = 1.0) *vs.* the second (M = 1.2, SD = 1.1, *p* = 0.040; M = 0.8, SD = 0.8, *p* < 0.001; M = 0.9, SD = 1.0, *p* < 0.001). In the group of primary care patients “fatigability” (M = 0.9, SD = 0.8), “irritability” (M = 0.9, SD = 1.0), and “sleep disturbance” (M = 0.8, SD = 0.9), achieved the highest mean scores, while in psychosomatic patients this was the case for “irritability” (M = 1.9, SD = 1.0), “sadness” (M = 1.8, SD = 0.9) and “work difficulty” (M = 1.8, SD = 0.9).

**Table 3:** The degree of orientation towards culture of origin (CO) and towards the host culture (HC), as well as depression levels according to gender, migrant generation, and setting.

Sub-Groups	Orientation towards Culture of Origin (CO), Mean (SD)	Orientation towards the Host Culture (HC), Mean (SD)	Depressive Symptoms (BDI), Mean (SD)
Women	45.7 (8.7)	32.2 (10.2)	21.8 (13.3)
Men	44.8 (8.0)	32.3 (8.9)	15.1 (14.0)
First migration generation (born in Turkey)	46.6 (8.3)	31.0 (9.6)	20.2 (14.1)
Second migration generation (born in Germany)	41.3 (7.4)	36.2 (8.8)	14.0 (12.9)
Primary care patients of Turkish origin	45.0 (8.0)	33.6 (8.8)	10.9 (9.7)
Psychosomatic outpatients of Turkish origin	45.6 (8.8)	30.6 (10.3)	28.2 (12.5)

### 3.5. Correlations between Acculturation Indices, Depressive Symptoms, Socio-Demographic, and Migration-Specific Variables

In the total sample, CO and HC were significantly associated with age, education level, migration generation, age at immigration, language proficiency, as well as with each other. Higher CO was significantly correlated with higher age (r = 0.195, *p* < 0.001), lower education level (r = −0.187, *p* < 0.001), belonging to the first migration generation (r = −0.295, *p* < 0.001), a higher age at immigration (r = 0.131, *p* < 0.014), less language proficiency (r = 0.369, *p* < 0.001) and lower HC (r = −0.335, *p* < 0.001). Higher HC was significantly associated with lower age (r = −0.187, *p* < 0.001), higher education level (r = 0.278, *p* < 0.001), belonging to the second migration generation (r = 0.238, *p* < 0.001), higher language proficiency (r = −0.429, *p* < 0.001), and younger age at immigration (r = −0.173, *p* = 0.001). In addition, a significant negative correlation between HC and depressive symptoms (total BDI scores) could be demonstrated (r = −0.267, *p* < 0.001). Higher levels of depressive symptoms were significantly correlated with higher age (r = 0.132, *p* = 0.004), female gender (r = 0.275, *p* < 0.001), lower education level (r = −0.263, *p* < 0.001), lower income (r = −0.167, *p* = 0.001), belonging to the first migration generation (r = −0.203, *p* < 0.001), and less language skills (r = 0.308, *p* < 0.001).

### 3.6. Predictors of Severity of Depressive Symptoms

To examine the influence of the acculturation indices as well as socio-demographic and migration-related variables on the BDI total score of the total sample, step-wise multiple regression analyses were performed. A model (model 1) was calculated only for socio-demographic variables (gender, age, education level, and income) as predictors and the total BDI score as criterion variable (see Table 4). Education level (β = −0.275; *p* < 0.001) and gender (β = 0.185; *p* < 0.001) were found to be significant predictors: higher education and masculine gender were associated with lower degree of depressive symptoms. The explanation of variance was 12.7%.

In model 2, socio-demographic variables (see above) and migration-specific variables (migration generation, length of stay in Germany, age at immigration, language proficiency) were included as predictors. We found a better language proficiency (β = 0.234; *p* < 0.001), masculine gender (β = 0.168; *p* = 0.003), and a higher education level (β = −0.132; *p* = 0.026), to be significantly associated with decreased levels of depressive symptomatology. The explanation of variance of BDI scores was 14.5%.

In model 3, socio-demographic variables (see above), migration-specific variables (see above), and both acculturation indices (CO and HC), were included as predictors. Significant influence on decreased depressive symptoms was demonstrated by masculine gender (β = 0.207; *p* < 0.001), better language proficiency (β = 0.202; *p* = 0.002), higher HC (β = −0.198; *p* = 0.001), higher CO (β = −0.163; *p* = 0.005), and higher education level (β = −0.116; *p* = 0.047). The explanation of variance was 19.0%.

**Table 4:** Linear regression analyses of depressive symptoms (BDI) for the total sample (N = 471) by socio-demographic, migration-specific and acculturation-related variables.

Independent Variables	Significant Predictors	Beta	*p*-value	Adj. R^2^
**Socio-demographic variables (model 1):** gender, age, education level, income	education level	−0.275	<0.001	12.7%
	gender	0.185	<0.001	
**Socio-demographic and migration-specific variables (model 2):** gender, age, education level, income, migration generation, length of stay in Germany, age at immigration, language proficiency	language proficiency	0.234	<0.001	14.5%
	gender	0.168	0.003	
	education level	−0.132	0.026	
**Socio-demographic and migration-specific variables and both acculturation indices (model 3):** gender, age, education level, income, migration generation, length of stay in Germany, age at immigration, language proficiency, orientation towards culture of origin, orientation towards host culture	gender	0.207	<0.001	19.0%
	language proficiency	0.202	0.002	
	orientation towards host culture	−0.198	0.001	
	orientation towards culture of origin	−0.163	0.005	
	education level	−0.116	0.047	

## 4. Discussion

The main focus of the present study was to investigate the association between acculturation levels and strategies and depressive symptoms in Turkish immigrants living in Germany. The most important finding was the observation that a higher degree of acculturation to both the heritage and the host culture was associated with lower levels of depressive symptoms. This result is consistent with previous research reporting a significant relation between affiliation to the new society (often measured as linguistic acculturation) and depressive symptomatology [16,22,24], or other indicators of mental health [19]; as well as studies reporting a relation between maintenance of identification with the origin culture and depression or psychological distress [21].

A possible explanation for the observed pattern is the assumption that advanced adaptation to the new cultural context plays a protective role for mental health. Higher acculturated individuals may have more resources and skills (e.g., language proficiency) to successfully challenge the demands of living in a new country, such as finding satisfactory employment. As a result, a high adaptation level to the host society may decrease the effects of acculturation stress. Whereas less acculturated persons may experience, in a chronically stressful way, a kind of social defeat [19] due to a lack of adequate competence in managing life effectively in a new society. A prolonged experience of outsider status may contribute to the manifestation of depressive symptoms, as social defeat has been shown to be a stressor inducing depression [34]. On the other hand, strong links with the origin culture may provide a feeling of identity and belonging, ensuring social support from the ethnic community. There is strong empirical evidence that social support is a substantial factor protecting against psychological distress and health problems [35]. Not maintaining cultural identity may lead to rejection by the ethnic group and loss of supportive social networks, making the individual more vulnerable to psychosocial stressors. In collectivistic cultures such as the Turkish, where the common good is prioritized over individual interests, factors such as maintaining traditions and norms, as well as conformity of in-group members play an important role in being accepted and supported [36].

An important idea suggested in previous research is that the integrationist perspective, which supports the full participation of ethnic minorities in institutions of the dominant society as well as maintenance of cultural roots, should be encouraged by public policies [7]. However, the degree of adaptation to the new society depends not only on the migrants themselves but also on immigrant policies (e.g., assimilation or integration as the favored official national acculturation strategy) and on the attitudes of the autochthonous population towards the migrants [21]. As effective measures for improving the mental health status of the migrants, interventions for improvement of proficiency of the new language and preservation of the culture of origin are recommended [19].

The low explanation of variance in the regression analyses is worth mentioning: Although HC and CO demonstrated to be significant negative predictors of depressive symptoms, as well as masculine gender, better language proficiency, and higher education, these five predictors only explain 19.0% of the variance. Similar low explanations of variance have been reported, for example, in the above mentioned study of Merbach *et al.* [24]. It can be assumed that other important factors that were not assessed in this study such as discrimination [24], or a sense of coherence [37,38], may also have a crucial impact on depressive symptomatology.

The protective function of orientation to both the host and the origin culture is supported by another important result of our study concerning the impact of acculturation strategies on depressive symptoms. In line with Berry´s theory and empirical research [7,8], we found integration to be associated with the lowest levels of depressive symptomatology and marginalization with the highest. These findings are in accordance with the results of a meta-analysis on acculturation and mental health, based on data from 325 studies (N = 72,013 participants), which confirmed integration to be the most favorable acculturation strategy for mental health and marginalization to be the least favorable [9]. The high relevance of affiliation to both cultures is associated with mental health, whereas an absence of roots in both cultures—or feelings of alienation from both—has the most negative impact on health problems. The integration strategy as the most adaptive acculturation style reflects a bicultural identity and thus provides the opportunity of using two different social support systems—those of both the ethnic group and the host society.

Another important finding concerns the role of gender and generational status in relation to acculturation and levels of depressive symptoms. Gender did not influence the levels of CO and HC, but migration generation did. The first migration generation (*i.e.*, persons born in Turkey) showed significantly higher CO and lower HC than the second migration generation (*i.e.*, persons born in Germany). This could be explained by the fact that the second generation migrants were born and socialized into the new country, have had continual contact with the new culture and therefore have a higher orientation towards the HC than the first generation migrants. Similarly, the first generation migrants were born and socialized in their country of origin, which may explain their stronger ties to the CO. Higher severity of depressive symptoms in female immigrants is supported by evidence from research of the general population [27] and research among immigrants in particular [3,6], and could be determined by factors such as a higher vulnerability for mental disorders, a higher acculturation stress, and a worse socio-economic situation compared to men.

The higher level of depressive symptoms in the first generation compared to second generation immigrants found in the present study is consistent with other investigations [6], while other studies have reported different findings supporting the immigrant paradox [39]. Many significant losses (e.g., of social contacts and support, accustomed surroundings, and often loss of profession and social status) and higher acculturation stress (e.g., learning a new language, searching for a new job) may be responsible for the poorer mental health in first generation immigrants, because they face more of these challenges than the second generation who grew up in the new country.

### Strengths and Limitations

To the best of our knowledge, this study is the first to explore an association between the four acculturation strategies and depressive symptoms among the largest immigration group in Germany.

Our study does have some limitations. Firstly, the selected samples (two Turkish health-care utilization groups) do not allow generalizations to be made for the immigrant population in general or exclusively for Turkish immigrants. The cross-sectional study design presents a further limitation because it does not allow drawing causal conclusions concerning the influence of the variables measured. Prospective investigations are required including representative samples of the migrant populations from different cultures to explore the effects of acculturation on mental health. Another limitation is the overrepresentation of first generation immigrants, which may be regarded as a bias. Moreover, the acculturation strategies should be measured by means of a validated and transculturally adapted questionnaire. Furthermore, when evaluating socio-economic status, in addition to using household income as an indicator, the number of persons living in the household should also be considered. Finally, the influence of contextual factors moderating the acculturation, such as discrimination for example, should be taken into consideration in future studies.

## 5. Conclusions

Our findings show that within the context of post-migration acculturation, high levels of orientation towards both the host and heritage culture were found to have a protective value against depressive symptoms, in the two health-care utilization groups with a Turkish migration background. A balance between identification with traditions of the heritage culture and an openness for and acquiring the competences needed in the new society seems to be one way of achieving successful acculturation and thus promoting a good health status [11]. Immigrants should therefore be encouraged by policy and health-care services to acquire the necessary skills to function well in the host culture, and to integrate their cultural roots, so that they develop a bicultural identity or a feeling of being comfortable within two cultures. Public mental health care institutions should favor the integration approach to acculturation of migrants. Accordingly, the focus should not only be on assimilation of migrants toward the host society but also on appreciating their client’s cultural heritage and working toward helping them to cope successfully within both cultures. From the integration perspective, it is also advisable that any suggestions to migrants should be culturally sensitive. The improvement of adaptation of migrants will require investigation of their specific cultural characteristics (e.g., values, attitudes *etc.*) in order to develop adequate prevention and intervention programs. Further research is indicated to provide information on the mechanisms underlying the association between acculturation and mental health.
